# Molecular docking analysis of melamine with nuclear factor erythroid 2-related factor 2 and succinate dehydrogenase

**DOI:** 10.6026/97320630018718

**Published:** 2022-08-31

**Authors:** Nitish Rai, Sheemona Chowdhary, Deepak Kumar, Rajasri Bhattacharyya, Dibyajyoti Banerjee

**Affiliations:** 1 Department of Biotechnology, Mohanlal Sukhadia University, Udaipur, Rajasthan, India; 2 Department of Experimental Medicine and Biotechnology, PGIMER, Chandigarh 160012, India

## Abstract

Melamine consumption causes oxidative stress by an unknown mechanism. Therefore, it is of interest to analyze the interaction of melamine with two important proteins involved in oxidative stress biology namely, nuclear factor erythroid 2-related factor 2 and succinate dehydrogenase. The molecular docking data shows the melamine binding with these two proteins at critical residues. These interactions can be logically perceived for the causation of melamine induced oxidative stress.

## Background:

Melamine or 1, 3, 5-triazine-2, 4, 6-triamine is a commonly used food adulterant. It causes a false-positive test for protein, which is used for adulteration of milk to falsely inflate the protein content [1]. The world has witnessed several melamine associated disorder outbreaks not leaving even children [2]. Melamine causes nephropathy, and melamine exposure can affect almost all the human body systems [3]. Melamine exposure causes inflammation and oxidative stress, which has the potential to affect multiple systems of the human body [4]. Guo et al. showed that melamine activates the NF-κB/cox-2 and NOX/ROS pathways that induce inflammation and oxidative stress highlighting the significant role of NOX in melamine-induced ROS formation [5]. A recent study showed the effect of single-nucleotide polymorphisms (SNPs) in Manganese superoxide dismutase (MnSOD) enzyme which could modify the protective response of antioxidant enzyme toward melamine induced oxidative stress and increases the risk of renal tubular injury in calcium urolithiasis patients [6]. Another study investigated the effects of melamine containing diets on the various parameters of Oreochromisniloticus and showed that melamine significantly reduced the activity of superoxide dismutase and glutathione peroxidase [7]. Nuclear factor–erythroid 2-related factor 2 (Nrf2) regulates the expression of more than 250 antioxidant enzymes, including glutathione peroxidase, heme oxygenase-1 (HO-1), and glutamate-cysteine ligase [8]. Nrf2 has been associated with different oxidative stress associated pathologies like renal disease, obesity, neurodegeneration, diabetes mellitus, atherosclerosis, and hypertension [9]. So presently, the Nrf2 signalling pathway is considered an important target against oxidative stress associated conditions. Further, Succinate dehydrogenase (SDH) is a mitochondrial electron transport chain component. It is observed that modulating SDH activity results in a change in ROS production. There is a decrease in ROS production when SDH activity is inhibited by malonate [10]. Our laboratory had earlier proposed the urinary melamine as a parameter of melamine adulteration of food and developed a point of care test for melamine detection in human urine [11-12]. It is observed that even a low dose of melamine exposure may increase the biomarkers of oxidative stress, which can increase the risk of kidney damage. Nevertheless, nothing much is known about the mechanism of causation of oxidative stress in the presence of melamine [13].Therefore, it is of interest to document the molecular docking analysis of melamine with nuclear factor erythroid 2-related factor 2 and Succinate Dehydrogenase.

## Methods:

### Preparation of nuclear factor erythroid 2–related factor 2 (Nrf2) and succinate dehydrogenase structures:

The 3-dimensional predicted structure of Nrf2 was downloaded from AlphaFold (AF- Q16236-F1) as the structure of the complete sequence was not available in Protein Data Bank. The structure obtained from Alpha fold had folded leucine zipper domain overlapping with NLS. The 3-dimensional structure of succinate dehydrogenase (PDB: 6VAX) was downloaded from Protein Data Bank. Further, only chain A of succinate dehydrogenase was considered for docking. The other ligands were also removed from the protein.

### Preparation of melamine:

The 3-dimensional structure of melamine (CID: 7955) was downloaded from the PubChem database.

### Molecular docking using AutoDock:

Molecular docking predicts the binding affinity of ligands with biomolecules. In the present work, we aimed to understand whether any interaction exists between melamine and the chosen proteins.

The docking of melamine with NRF2 and succinate dehydrogenase was performed using AutoDock (v 4.2.6). It is known that the leucine zipper and the basic region of NRF2 (present in the Neh 1 domain) are necessary for DNA binding and association with dimerization proteins for the induction of antioxidant response in case of oxidative stress [14-15]. Therefore, residues in Neh 1 domain were targeted for docking with melamine. The residues considered were Arg499, Lys506, Lys516, Glu524, His551, and Glu556. In case of succinate dehydrogenase, the binding sites considered for docking were chosen from UniProt (ID: P31040). The protein and the ligand preparation were performed using AutoDock Tools. The functions like adding H-atoms, computing gasteiger charges, merging non-polar hydrogen and assigning atom type (AD4 type) were performed for protein preparation. For the ligand preparation, the number of torsions was set. The prepared protein and ligand files were saved in PDBQT format. AutoGrid was used to prepare the grid map using a grid box. The dimensions of the docking grid were 60 Å x 60 Å x 60 Å. The grid centre was defined as per the residues chosen in each protein for docking (His 551, ND1). During docking, the protein and ligand were considered rigid. The Lamarckian genetic algorithm was used to predict melamine binding with the proteins. The number of GA runs was set as 60, and the rest of the parameters were kept at default. Therefore, 60 conformations of the ligand were generated with associated energies. The results were analyzed by clustering analysis. The lowest (maximum negative) energy conformation from the largest cluster was chosen to represent the binding mode of the ligand with the protein.

## Results:

### Docking studies with NRF2:

The binding of melamine was observed considering all the targeted residues participated in hydrogen bonds, hydrophobic interactions, etc. (Figure 1 - see PDF). However, certain docked conformations of the targeted residues showed higher binding energy (Table 1 - see PDF). Higher binding energy indicates a stronger binding affinity of melamine to NRF2 protein. The docked conformation of melamine-NRF2 where Lys516 (-7.01 kcal/mol) residue in the basic region of zipper domain was targeted, showed binding near the residues of DLG motif (Figure 1C - see PDF).The DLG motif is critical for binding with Keap-1. Furthermore, the highest energy docked conformation (-7.29 kcal/mol) where Glu524 was targeted showed binding with the residues of the zipper domain (Figure 1D - see PDF). These results suggest that melamine shows an affinity for binding with the zipper domain and DLG domain. In case of other residues targeted, binding was observed outside the zipper and DLG domain. The clustering analysis showed that the highest energy clusters (asymp;-7.0kcal/mol) consisted of almost 100% of the bound melamine conformations considering Lys516 and Glu524 as grid centers (Table 1 - see PDF). It indicates that based on the structure of NRF2, the binding orientation may be varied at the target site.

### Docking studies with succinate dehydrogenase:

The interaction of melamine with succinate dehydrogenase was studied and compared with other ligands (succinate and malonate) to understand its binding characteristics (Table 1, Figures 2-4 - see PDF). Succinate, a natural substrate of succinate dehydrogenase, was docked with succinate dehydrogenase, considering His296 as the grid centre (the reason for grid centre is written below). The binding energy was -2.35 kcal/mol in the largest cluster. Malonate is a known competitive inhibitor of succinate dehydrogenase; therefore, its interaction with one of the substrate-binding sites (His 296) was also studied. We observed that malonate showed binding energy (-2.14 kcal/mol) with succinate dehydrogenase, comparable to the succinate-succinate dehydrogenase complex. The melamine's binding site(s) in succinate dehydrogenase is not known. Therefore, different binding sites were selected as grid centres for docking with melamine based on the literature. The selected grid centres were His296, Thr308, Arg340, His407 and Arg451 (Table 1 - see PDF). Although different grid centres were selected in most cases, melamine binds to the same residues, and only bond distance varied (Figure 2A, C, E - see PDF). In two cases, energy was more negative, and the binding orientation differed (Table 1 - see PDF). Based on the clustering analysis, two types of clusters were observed (≈ -5.2 and -6.3 kcal/mol) (Table 1 - see PDF).

Both clusters consisted of ≈ 90% of the conformations irrespective of consideration of grid centre. It indicates that melamine has a stronger affinity than malonate for binding with succinate dehydrogenase in terms of binding energy. Next, we wanted to understand whether melamine can bind with succinate dehydrogenase bound succinate complex and vice versa. Melamine was docked with succinate bound succinate dehydrogenase, and succinate was docked with melamine bound succinate dehydrogenase, considering His296 as the grid centre (Figure 3 - see PDF). We observed that the binding affinity of both the ligands (melamine and succinate) with succinate dehydrogenase is more favorable than succinate dehydrogenase complexed with succinate alone. The binding energy was observed to be higher in both the cases (-5.71 and -5.3 kcal/mol) in comparison to succinate dehydrogenase complexed with succinate only (-2.35 kcal/mol), but comparable with melamine only bound succinate dehydrogenase complex (-5.30kcal/mol). Similarly, in succinate bound succinate dehydrogenase complex, malonate was docked. We observed that malonate showed less favorable energy in comparison (-1.1 kcal/mol) to succinate docked complex (-2.35 kcal/mol) (Figure 4 - see PDF). It indicates that malonate may not be able to bind with succinate dehydrogenase when succinate is already bound to the enzyme. Furthermore, in malonate bound complex, succinate was docked. We observed that succinate showed more favorable binding energy (-2.02 kcal/mol) in the malonate docked complex in comparison to the succinate bound complex where malonate was docked (-1.1 kcal/mol) (Table 1 - see PDF). It indicates that succinate has more binding affinity for succinate dehydrogenase than malonate.

## Discussion:

Oxidative stress is one of the major attributes of melamine associated toxicity [16-20]. A study found the presence of mitochondrial vacuoles in the injured proximal tubular cells of rats at 0.5h post-exposure to melamine and cyanuric acid (MCA) mixture crystals to induce acute renal toxicity. This showed that the crystal could induce physical injury to the proximal tubular cell membrane, resulting in cell degeneration [21]. However, there is a lack of studies explaining the molecular mechanism of melamine-induced mitochondrial dysfunction. In the present work, an in-silico study is performed to analyze the molecular interaction of melamine with Nrf-2 and Succinate Dehydrogenase to explain the mechanism underlying melamine induced oxidative stress and mitochondrial dysfunction. The human Nrf2 protein contains 605 amino acids and seven highly conserved regions designated as Nrf2-ECH homology (Neh) domains. The role of Neh domains includes hetero dimerization, trans-activation, and interaction with other proteins and receptors [22]. When Nrf2 binds with the antioxidant response element of the DNA and expresses certain cyto protective genes that are responsible for the antioxidant response, therefore, if Nrf2 binds with some small molecule like melamine, this critical function may be hampered. The in-silico analysis showed that melamine binds to Nrf-2 in the region of the zipper domain and DLG motifs present in the Neh2 domain. The DLG motif is explicitly known to bind with Keap1. Under typical scenario, Keap1 forms a Cullin–RING E3 ligase complex by interacting with the Cul3 protein to degrade Nrf2. Oxidative stress inactivates Keap1 to accumulate high levels of Nrf2 protein to express the critical stress response genes. The binding of melamine may inhibit the degradation and DNA binding ability of Nrf-2 and attenuated response against oxidative stress. Human Succinate dehydrogenase produces ROS. Its inhibition is proved to generate less ROS [23,24]. Human Succinate dehydrogenase possesses four structurally different subunits. Two subunits, SdhA and SdhB, form a hydrophilic head having enzymatic activity and the other two subunits, SdhC and SdhD, have a hydrophobic membrane anchoring role. Sdh A is a flavor protein with two isoforms, and SdhB is an iron-sulfur protein [25]. Molecular docking was performed to investigate the binding pose of melamine with succinate dehydrogenase. Results are shown in Table 1, and Figure 4(see PDF) shows that succinate binding with SDH is more favorable than malonate in terms of binding energy. This explains the fact that malonate is a competitive inhibitor of SDH. Further, melamine (Melamine-SDH complex) favoured the succinate binding with SDH in terms of binding energy (Table 1 - see PDF). All of these point toward the fact that melamine will cause the SDH to act more efficiently, causing hyper functioning of the electron transport chain and thus generating more ROS. This phenomenon can cause oxidative stress; particularly if Nrf2 induced antioxidant defense system is under expressed. We present this concept in a line diagram for ease of understanding the readers (Figure 5 - see PDF). Following melamine exposure, oxidative stress is reported. However, here we propose a mechanism for the genesis ofoxidative stress following melamine exposure.

## Conclusions:

Our in-silico study showed that melamine could bind with Nrf2 and succinate dehydrogenase with certain features from which we can explain how oxidative stress can happen following melamine exposure. Since melamine adulteration of food (milk) affects even children, we believe that issues concerning melamine should be subjected to focus research urgently. We recommend experimental verification of our proposed concept.
